# Physical Diagnosis as Related to Dental College Curricula

**Published:** 1902-08

**Authors:** A. H. Peck

**Affiliations:** Chicago


### PHYSICAL DIAGNOSIS AS RELATED TO DENTAL COLLEGE CURRICULA.[1]

BY A. H. PECK, M.D., D.D.S., CHICAGO.

In view of the fact that during the past two years a number of the State Boards of Dental Examiners throughout the country have added the subject of *physical diagnosis* to their list of studies which must be passed to secure a license to practise dentistry in their respective States; also that I have for some years been impressed with the desirability and, I may say, the necessity of adding this subject to our dental college curricula, I concluded this would be as fit a subject as any for my paper at this time.

Heretofore this subject, whenever taught at all, has received what I may term unconscious attention from various teachers,—that is to say, in the regular teaching of their departments they have naturally referred to phases of physical diagnosis, but not until the past year has the subject been made a separate department and a complete course of instruction given.

The knowledge of man has steadily increased, keeping apace with civilization. Man has been brought to a higher plane through scientific investigation; his mind broadened and ripened in the fields of research. The furtherance of their profession and the elevation of their fellow-men have ever been uppermost in the minds of the great men of the past.

As you are familiar, it was discovered ages and ages ago that teeth were filled with pieces of wood, ivory, and other materials, as evidenced by the researches of the catacombs of Rome and Naples, thus assuring us that the art of preserving teeth was known to our ancestors of those very early times. Unfortunately, however, we do not know who the great dentists of those ages were, as the records of this work have been lost. Had this work been entirely satisfactory to these dentists and their patients, the latter being pleased and contented with such operations, our profession would never have attained to its present high, enviable position in this professional world.

Dissatisfaction with prevailing methods, and the laudable

---

[1] Read before the Section on Stomatology, American Medical Association, Saratoga Springs, June 10, 1902.

desire to excel, set men to thinking and to doing, the result being that dentistry has developed from an humble trade to an honored profession, affording a field for usefulness to thousands, and whereby the suffering of the entire civilized world can be and is, in a great measure, alleviated.

During the past few years the courses of study have been lengthened in all professional schools. Only a short time has elapsed since a medical student could graduate after attending two courses of instruction of six months each, but now one is required to attend four years of nine months each, after having gained a good scientific or classical education as a foundation upon which to build his professional knowledge, thus requiring from six to eight years of college work to receive his degree of Doctor of Medicine. Let us not forget that dentistry has by no means been slumbering all the while, for the educational requirements for admission to a dental college have been steadily advancing, and the number of years and length of terms increasing, until now schooling equal to the second year of high school work is required, and another year four years of seven months each will be required.

Only a few years ago a student received a few lectures on physiology, when that part of his course was considered finished, but now it is one of the most important branches he has. Histology, pathology, and bacteriology have become important subjects, and the dentist would also be considered very lame without a knowledge of anæsthesia and oral surgery. More than this, the amount of practical work that must be done as a part of the dentist's preliminary education has more than doubled. What is the object of this advance? It is that his knowledge may be broadened, extended, and that he may be placed on a higher plane with mankind, as well as that he may be better able to satisfy himself and his patients after engaging in the practice of his chosen profession.

We, as professional men, are continually being called upon to give opinion as to the etiology and prognosis of certain diseases, and who will attempt to gainsay the statement that this we should be not only willing, but able to do, and it is imperative that we be as nearly correct in such counsel as possible. At times we find this easy, and again it taxes us to the limit, if not beyond; all of our knowledge and reason is called into play before we are able to make definite statements.

It is not always an easy matter to tell just how much vitality a patient has, nor how much of a nervous shock one can endure, nor how long one can remain in a dental chair at a sitting without sustaining material injury. This we, as dentists, should know, so that our patients, on leaving our offices, will have received professional benefit instead of injury.

Who of you have not seen or are not cognizant of neurotic patients who were nervous wrecks for days after having had a large amount of dental work done? With the requisite knowledge and the exercise of forethought and judgment all this can be avoided. A few more sittings of shorter duration would have completed the work, at the same time acting as a stimulus rather than as a nervous shock. This knowledge we can gain only by a thorough study of our patients, and an understanding of the cause or causes of their ailments.

I hope to see the time when a dentist will inquire into the health and symptoms of his patients before deciding on the amount of work that is proper and safe to be done at any one sitting, as should a physician before prescribing a certain amount of a drug or drugs that are to be given for an ailment of the patient.

To judiciously outline our work we, as dentists, must have as thorough a knowledge as possible of the various diseases of mankind, especially those affecting the vital organs or those organs most likely to suffer when shock is inflicted. The symptoms of these diseases, also the physiological changes that may occur, are necessary to be understood. Who of us would keep a patient afflicted with organic heart disease in our chair for an unusually long and fatiguing operation if we were able to inform ourselves of the true condition of these parts?

There is only one way for us to gain this knowledge, and that is for us to familiarize ourselves with the normal heart, as to location, size, beat, rhythm, and sounds, thus enabling us to recognize pathological conditions when present. How embarrassing it must be for any dentist, after advising the administration of a general or local anæsthetic, to be told, on consulting the family physician, that such a course would mean certain death to the patient, whether true or not.

Physical diagnosis is the term used to designate those methods which are employed in the detection of disease during life by the anatomical changes produced by it. The nature and extent of such changes can only be recognized and appreciated by the divergence which they cause in the affected organs from the known physical condition of these organs when in health.

The significance of physical signs in disease cannot be determined by theory; only by clinical observation confirmed by observation after death can this significance be determined.

If it be granted that it is at all desirable that the dentist shall possess this knowledge I am talking about, it at once becomes evident that he must enter into a systematic and thorough study of the only methods by which these physical signs can be determined in the living subject, and these methods are: 1. Inspection. 2. Palpation. 3. Mensuration. 4. Percussion. 5. Auscultation. 6. Radioscopy.

Some of these methods have been in use for many centuries. Palpation, for instance, was used in the Neolithic or polished stone age, 1500 B.C., to demonstrate the presence of fluctuation, while radioscopy is practically new. This method is the outcome of the discovery of the X-ray, by which, with the use of the fluoroscope, tumors or solid bodies are located in various parts of the body, that were impossible of discovery before. Fractures of bones, the exact kind and position, are determined by looking at the bone direct. Tumors of the internal organs are observed by this means, thus enabling one to diagnose conditions which were impossible of discovery before the X-ray was in use.

One must also be conversant with the various areas into which the body is divided, and which are bounded by definite anatomical relations. This is necessary, that one being familar with the normal size and location of an organ can determine whether it is in its proper position.

It is necessary to know that the first area from a physiological stand-point is the *supraclavicular* region; and that this area is definitely bounded below by the inner three-fifths of the clavicle, internally by the trachea, and superiorly by a line extending from the junction of the outer with the middle third of the clavicle to the top of the trachea. Also, it is necessary to know that normally within this area are to be found the apex of the lung, the carotid artery, the subclavian artery, the subclavian vein, and the jugular vein.

Next below this is the *clavicular* region, which is that part of the thoracic cavity lying back of the inner three-fifths of the clavicle. An understanding of the anatomical boundaries and contents of this region is also necessary, but with which I shall not inflict you in this paper.

The most important regions, from the stand-point of the dental practitioner, are the following: *Infraclavicular,* the boundaries of which must be carefully studied, that one may recognize the presence of the vital anatomical structures and organs in their normal positions. In this region are to be found, on the right side, lung tissue, the ascending vena cava, the right bronchial tube lying back of the sternocostal articulation, and also a small portion of the arch of the aorta. On the left side are found the pulmonary artery from its origin to its bifurcation, the left bronchial tube lying a little below the second sternocostal articulation.

The next region of special importance to the dentist, and which lies immediately below the preceding one, is called the *mammary* region. The lowest region in the anterior aspect of the thoracic cavity is called the *inferior mammary.*

Centrally located is the sternum, this area being divided into three regions: (1) the *suprasternal;* (2) the *upper sternal;* and (3) the *lower sternal.*

The back is divided into three regions: (1) the *suprascapular;* (2) the *infrascapular;* and (3) *the interscapular.*

All these regions should be carefully studied, as indicated above, in the two instances in which the boundaries and contents are stated.

A knowledge of the size and exact location of the heart are especially important, in the average subject the base of this organ being found at the second intercostal space, the apex-beat or the maximum impulse being at the fifth intercostal space from three-fourths of an inch to an inch to the left of the sternum. It must be understood that the apex-beat does not locate the apex of the heart, the latter being about an inch to the left of the beat.

The anatomy of the heart must be studied. It is necessary to know that there are four different valves, and what is expected of them in the performance of their normal function, and that the positions on the chest where the sounds made by the valves can be most distinctly heard are not immediately over the organ.

I thus briefly outline this foundation work that there may be no mistake as to what I consider necessary in the schooling of prospective dentists, that they may be able intelligently to apply the six methods of eliciting the physical signs of the various pathological conditions of those diseased organs bearing directly on the practice of dentistry.

It is also necessary to be thoroughly conversant with the meaning of these various methods of physical diagnosis, how each is to be employed, and what is to be learned by it. That *inspection* means only that which can be determined by looking at the patient without further means of diagnosis. That *palpation* means the examination of the parts by the laying on of the hands, and in this method that only the tips of the fingers may be used, or the palms of the hands as a whole. That with *mensuration* certain facts are to be determined by the process of measuring. That by *percussion* is meant the tapping of the chest to ellicit certain sounds under the varying conditions; that there are different methods of percussion, the *immediate* and the *mediate.* That *auscultation* is the act of listening for sounds within the body, chiefly to ascertain the condition of the lungs, heart, pleura, and other organs; that there are different methods of auscultation,—the *immediate,* which is the application of the ear directly to the part, and the *mediate,* which is by use of the stethoscope.

The *pulse* is such an accurate index to many of the lesions of the heart, it is necessary that one shall understand it in all its variations.

Thus would I have dental students instructed. I trust this paper will receive full and unrestricted discussion, for I want to know whether, in your judgment, this branch should be added to the curriculum of our dental institutions of learning.

This is a subject that has engaged my attention for some time, and it was my desire more than two years ago to present this subject to the profession, and urge its teaching in our schools, but, listening to the advice of trusted friends that the time was not ripe for it, I desisted. During the past year it has been taught in the institution with which I am connected, and, I believe, also in one other college. As I see it now, I cannot understand how any one can advise otherwise.

I hope to see prospective dentists so instructed in the future that they shall be able to recognize diseased conditions of at least these vital organs, and thus be enabled to avoid serious and possibly fatal mistakes. When this knowledge is acquired and successfully practised, the dentist at once gains the implicit confidence of his patients, his word with them becomes law, and his opinion is sought and respected. Such a dentist is a real benefactor in the community in which he resides, and his success is assured.

He also has the satisfaction of knowing he is one who has participated in that "higher education," the practice of which can only result in assisting to elevate the standard of his profession, and to place it on a higher plane in its relation to other progressive professions.

